# Association of Bicipital Peritendinous Effusion with Subacromial Impingement: A Dynamic Ultrasonographic Study of 337 Shoulders

**DOI:** 10.1038/srep38943

**Published:** 2016-12-12

**Authors:** Ke-Vin Chang, Wei-Ting Wu, Levent Özçakar

**Affiliations:** 1 Department of Physical Medicine and Rehabilitation and Community and Geriatric Research Center, National Taiwan University Hospital, Bei-Hu Branch and National Taiwan University College of Medicine, Taipei, Taiwan; 2 Department of Physical and Rehabilitation Medicine Hacettepe University Medical School, Ankara, Turkey

## Abstract

Bicipital peritendinous effusion (BPE) is the most common biceps tendon abnormality and can be related to various shoulder ultrasonographic findings. Since the association of BPE with subacromial impingement is unclear, our study aimed to explore its association with the dynamic subacromial impingement test during ultrasound (US) imaging. We included 337 shoulders referred for US examinations and quantified the amount of BPE. Effusion more than 1 mm in thickness was considered a positive finding. A comparison of three grades of subacromial impingement, adjusted by patient demographics, static sonographic shoulder pathology, and physical findings, by using multivariate regression models revealed that the odds ratio of subacromial impingement (with 95% confidence intervals) in the presence of BPE was 6.54 (3.21–13.32) in grade 1, 6.93 (3.05–15.76) in grade 2 and 3.18 (1.48–6.80) in grade 3. An increase in age, subdeltoid bursitis, full-thickness supraspinatus tendon tear, and shoulder stiffness were also associated with BPE. Since our study demonstrated a positive association of BPE with all grades of impingement, a US dynamic subacromial impingement test is suggested when BPE is present. Future prospective studies are needed to identify changes in BPE after treatment.

The subacromial space is bordered by the undersurface of the acromion, coracoacromial ligament, and acromioclavicular joint superiorly and the humeral head inferiorly^1^. The structures passing through the space are the supraspinatus and subscapularis tendons, the long head of the biceps tendon, and the subdeltoid bursa^2^. Variations in acromial morphology, superior translocation of the humeral head, and acromial depression due to aberrant scapular motion cause narrowing of the subacromial space and lead to impingement^3^. Findings of the subacromial impingement include the presence of acromio-clavicular osteoarthritis or calcific tendinosis on radiographs and acromial deformity, rotator cuff, and subacromial bursa abnormalities on magnetic resonance imaging^4^. However, these imaging modalities are unable to dynamically evaluate the shoulder joint, whereas high-definition ultrasound (US) can provide real-time visualization of components inside the subacromial space during shoulder motion.

The long head of the biceps tendon (LHBT), originating from the superior glenoid labrum and supraglenoid tubercle, is situated between the supraspinatus and subscapularis tendons inside the subacromial space and is vulnerable to impingement^5^. Bicipital peritendinous effusion (BPE) is commonplace during shoulder US evaluation and is attributed to intra-articular pathology because the bicipital tendon sheath derives from the extension of the glenohumeral joint capsule^6^,^7^. Several recent ultrasonographic studies have demonstrated independent associations of BPE with subscapularis tendinopathy and subdeltoid bursitis^8^,^9^,^10^. Since the subscapularis tendon and subdeltoid bursa have no direct connection with the glenohumeral joint, concomitant presence of BPE and subdeltoid bursitis or subscapularis tendinopathy may all result from the same pathological mechanism, namely subacromial impingement. Although the relationship between BPE and static sonographic images of shoulder joints was established in previous research^8^,^9^,^10^, its association with dynamic US examination has never been clarified. Therefore, the present study was designed to explore the association of the development of BPE with the US dynamic subacromial impingement test.

## Method

### Study population

The present study employed a cross-sectional design, and analyzed the clinical and sonographic data derived from patients referred for shoulder US examinations. The research was approved by the institutional review board of National Taiwan University Hospital (IRB NO. 20141210RINA) and the requirement for informed consent was waived because the project was conducted by reviewing an existing data bank of routine examinations. Since January 2013, an established referral sheet was required to complete before the examination. The form comprised side, duration, and type of shoulder pain, shoulder range of motion, and physical examination of the shoulders including bicipital groove tenderness, speed test, Yergason’s test, empty can test, Neer test, Hawkins-Kennedy test, painful arc test, and shoulder stiffness (defined as 50% limitation in the shoulder range of motions and used for a clinical diagnosis of adhesive capsulitis)^10^,^11^,^12^. We included patients over 20 years of age and excluded participants with a diagnosis of systemic rheumatologic diseases, malignancy, major injuries or operations on the examined shoulder, and those who had received injections within 6 months before US scanning.

### Protocol of shoulder ultrasound examination

All the US images were obtained by a board-certificated physiatrist with a 10-year experience in musculoskeletal US examination. During scanning of the LHBT, patients were seated with the elbow flexed and the shoulder adducted. The transducer was placed at the upmost level of the bicipital groove when obtaining the short-axis image of the biceps tendon. The long axis of the subscapularis tendon was visualized during shoulder external and internal rotation. The supraspinatus tendon and subscapularis bursa were scanned by placing the arm behind the back to shift the tendon away from the coverage of the acromion. The hand was put on the contralateral shoulder during the examination of the infraspinatus tendon and the posterior shoulder recess^8^,^9^,^10^,^13^. Regarding the dynamic subdeltoid imaging test; the arm was rested beside the trunk with the transducer placed lateral to the acromion along the scapular axis. The patient was then asked to raise their shoulder in the scapular plane to the horizontal level. Practices were allowed until the patient could finish the task^14^.

**Figure 1:**
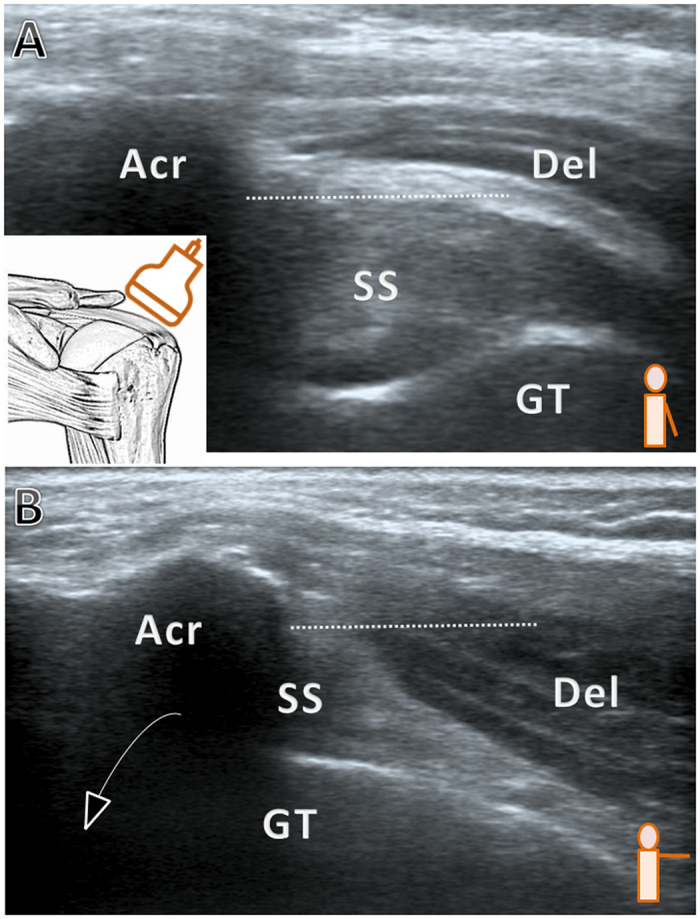
Grade 0, no impingement (**A**) The arm is rested beside the trunk (**B**) The arm is elevated to 90 degrees in the horizontal plane. The sonographic presentation of grade 1 impingement is the same as that of grade 0 but with pain during shoulder elevation. Acr, acromion; Del, deltoid; GT, greater tuberosity; SS, supraspinatus tendon; dashed line: the inferior edge of the acromion.

**Figure 2:**
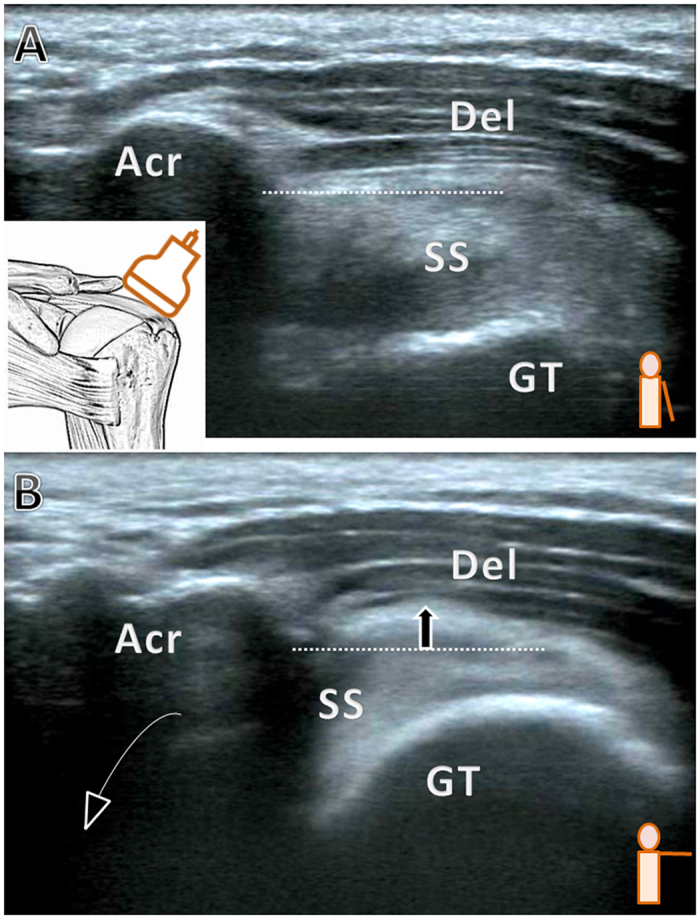
Grade 2, pain during shoulder elevation with soft tissue impingement. (**A**) The arm is rested beside the trunk. (**B**) The arm is elevated to 90 degrees in the horizontal plane. Acr, acromion; Del, deltoid; GT, greater tuberosity; SS, supraspinatus tendon; dashed line: the inferior edge of the acromion; arrow: bulging soft tissue.

**Figure 3:**
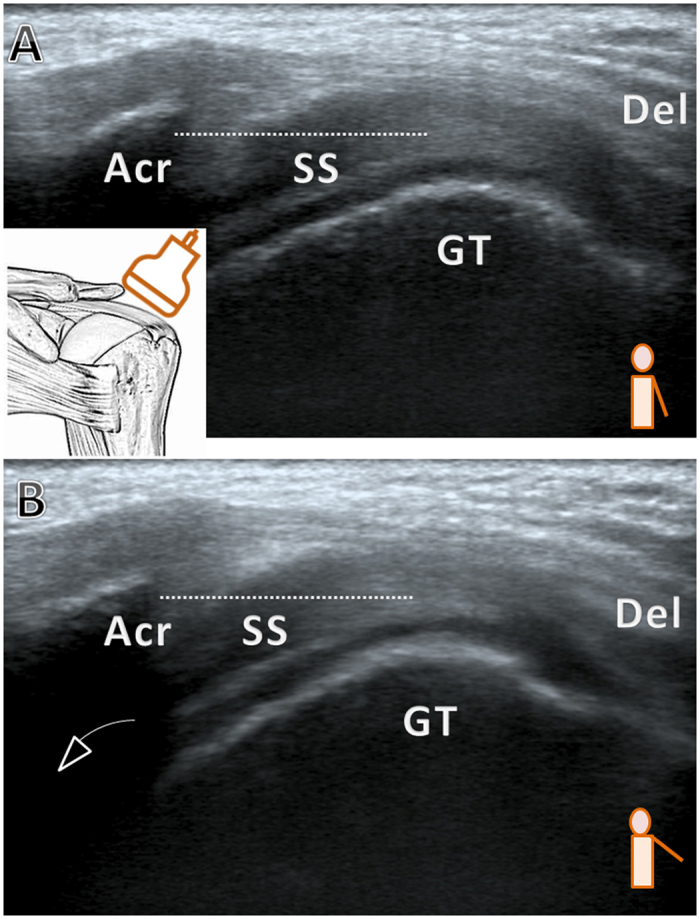
Grade 3, pain during shoulder elevation with upward migration of the humeral head that then fails to pass beneath the acromion. (**A**) The arm is rested beside the trunk (**B**) The arm is elevated to 45 degrees in the horizontal plane. Acr, acromion; Del, deltoid; GT, greater tuberosity; SS, supraspinatus tendon; dashed line: the inferior edge of the acromion.

Ultrasonographic diagnosis of tendinopathy comprised hypoechogenicity and swelling of the involved tendon. Hyperechoic plaques with or without acoustic shadowing signified tendon calcification. Subluxation of the biceps tendon was diagnosed if part of the tendon was displaced from the bicipital groove, whereas dislocation was diagnosed if the whole tendon was outside the groove. A full-thickness tear of the rotator cuff tendons was defined as an intra-tendinous gap extending from the bursal to the articular aspect, while a partial thickness tear was defined as a gap on either side or inside the tendon^8^,^9^,^10^,^13^,^15^. One antecedent study indicated that simultaneous presentation of BPE with subdeltoid bursa effusion usually coexisted with rotator cuff tendon tears^16^. In order not to let this phenomenon confound our result, the variable, subdeltoid bursitis (defined by fluid collection or hypoechoic synovium more than 2 mm in thickness) would be used in the regression model. The dynamic subacromial impingement test identified 4 grades of impingement based on previous studies: grade 0, no impingement (Fig. 1); grade 1, pain during shoulder elevation without soft tissue impingement; grade 2, pain during shoulder elevation with soft tissue impingement (Fig. 2) and grade 3, pain during shoulder elevation with upward migration of humeral head which then failed to pass underneath the acromion (Fig. 3)^14^. The grading system was based on the dynamic sonographic findings which were not completely the same with what is defined as rotator cuff impingement defined by MR arthrography^17^.

### Measurement of the bicipital peritendinous effusion

**Figure 4:**
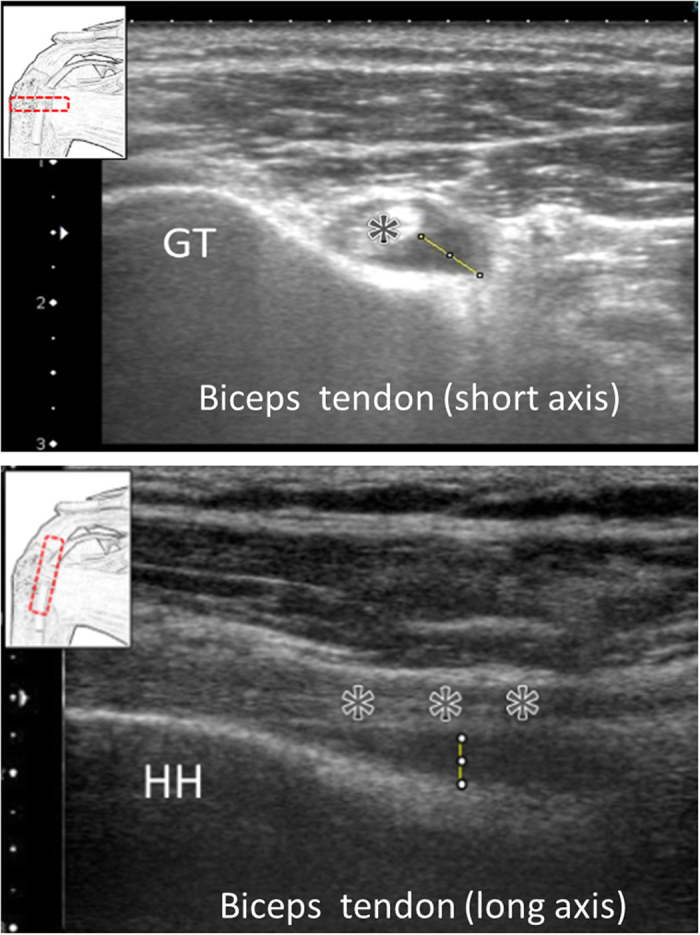
Quantification of the thickness of the bicipital peritendinous effusion. Asterisk: biceps tendon; GT: greater tuberosity; HH: humeral head; caliper: pertendinous effusion.

The short axis view of the biceps tendon (with light touch of the probe) was used for measurement of the BPE, which was visualized as an anechoic compressible fluid collection surrounding the LHBT^8^,^9^,^10^,^13^. A point on the border of the biceps tendon with the greatest effusion thickness was determined visually. A line was drawn perpendicular to the tangential plane passing through the visually determined point until the line reached the parietal layer of the tendon sheath. The distance between the points on the tendon sheath and the biceps tendon border was used as the maximal thickness of BPE. Most of the measurements were performed in the short axis view of the biceps tendon. If the effusion was better seen in the long axis view, its thickness was determined on the basis of the maximal thickness between the inferior border of the tendon and the tendon sheath closest to the floor of the bicipital groove (Fig. 4). The software Image J (National Institutes of Health, 9000 Rockville Pike, Bethesda, MD 20892) was used to conduct the measurements. Several previous researchers showed that using this method to quantify BPE, the correlation coefficients of inter- and intra-observer reliability can be up to 0.93 and 0.76, respectively^8^,^9^,^10^,^13^. One previous study showed that effusion thickness more than 1 mm was associated with various sonographic pathologies of the shoulder^10^. Sensitivity and specificity values as regards BPE in predicting rotator cuff lesions have also been verified by the same research group^8^. As such, in accordance with the recent/relevant literature, effusion thickness of more than 1 mm was used as the cut-off value to define physiological and pathological BPE^8^,^10^.

### Statistical analysis

Based on the previous studies, the ratio of patients with and without BPE was assumed to be 2 in the general population^8^,^9^,^10^,^13^,^18^,^19^. The proportion of positive subdeltoid impingement tests during dynamic US examination was estimated to be 40% in the group with effusion and 25% in those without effusion. In order to achieve a power of 80% with an alpha value under 0.05, more than 233 participants would be required.

The Shapiro–Wilk test was used to examine whether the distribution of continuous variables fit the assumption of normality. If the assumption was met, univariate analysis was performed by using Student’s t test; otherwise, the *Mann*–*Whitney* U test was used. In terms of categorical variables, the Chi-square test was used to examine differences between groups. Multivariate logistic regression was used to explore the relationship between BPE and the sonographic dynamic subacromial impingement test, which was expressed by using odds ratios (ORs) and 95% confidence intervals (CIs). The descriptive variables in Model 1 included solely the sonographic dynamic subacromial impingement test, whereas Model 2 added static sonographic findings and Model 3 further added physical findings. The analysis was performed by using SAS (Version 9.2, SAS Institute, Cary, NC, USA) and a *p* value ≤ 0.05 was considered statistically significant.

## Results

Three hundred seventy five US examinations that had been performed between January 2013 and December 2015 met the inclusion criteria. After discarding 32 sets of repeated examinations and 5 sets of unclear biceps images, 337 shoulders were included for quantifying the thickness of BPE, present in 146 shoulders (43.3%). The univariate analysis of basic demographics revealed that the group with BPE was older but did not have significant differences in proportions of genders or affected sides. Dynamic impingement, subdeltoid bursitis, supraspinatus tendon full thickness tear, supraspinatus bursal-sided partial thickness tear, and infraspinatus tendon tear were more prevalent in shoulders with BPE. More positive findings of the empty can test, painful arc test, and shoulder stiffness were seen in the BPE group (Table 1).

In the multivariable regression analysis for Model 1, all grades of dynamic subacromial impingement were associated with BPE after adjusting for age, gender, and affected side. ORs and their 95% CIs were 6.67 (3.50–12.73) for grade 1, 7.45 (3.49–15.90) for grade 2, and 3.74 (1.97–7.10) for grade 3. The association between BPE and various types of dynamic subacromial impingement remained significant without shifting of the point estimates of ORs following inclusion of static sonographic findings in Model 2 or by the inclusion of physical findings in Model 3. BPE was also associated with subdeltoid bursitis [OR: 3.30 (1.84–5.93) in Model 2 and OR: 3.42 (1.88–6.24) in Model 3] and supraspinatus tendon full thickness tear [OR: 4.28 (1.56–11.74) in Model 2 and OR: 4.63 (1.62–13.21) in Model 3]. Shoulder stiffness was the only physical finding associated with BPE [OR: 1.89 (1.02–3.51) in Model 3] (Table 2).

## Discussion

To the best of our knowledge, this is the first study to explore the relationship between sonographic subacromial impingement and BPE, the most common pathology of the LHBT. Our results showed that all grades of subacromial impingement were associated with BPE, and the association remained after adjusting for concurrent sonographic shoulder abnormalities and physical findings.

High resolution US has been widely used in diagnosing shoulder problems and demonstrates the ability to assess shoulder kinematics through dynamic tests^20^,^21^. Although static images for the subacromial space delineate the structural degeneration following impingement, only dynamic examinations in real time can recognize improper structural interactions during shoulder movements^14^. Further, a positive subacromial impingement test during dynamic evaluation may reveal rotator cuff abnormalities in a shoulder with normal static images. Likewise, inclusion of a dynamic subacromial impingement test could provide a better insight into understanding the cause of BPE developed in a painful shoulder.

During shoulder elevation, the supraspinatus tendon acts as an important humeral head depressor, allowing the proximal humerus to pass underneath the acromion^3^,^22^. The LHBT works synergistically with the supraspinatus tendon and is vulnerable to tensile stress with impingement^5^. Although BPE is considered to consist mainly of overflow fluid from the glenohumeral joint, several recent studies have proposed an alternative mechanism, namely over-secretion of synovial fluid against the increased friction in the bicipital tendon sheath^8^,^10^,^13^. Two findings in our data set support this assumption. First, supraspinatus tendon full thickness tears, the leading cause of increased fluid inside the glenohumeral joint, only accounted for 11.5% of our participants, in contrast with 43.3%, the prevalence of BPE (Table 1). Second, even in the lowest grades of dynamic aberrations, pain was elicited during shoulder motion without evidence of anatomic impingement and it, too, was associated with BPE. Moreover, the association was not accompanied by concurrent dynamic sonographic abnormalities.

When a shoulder develops grade 2 impingement, it indicates that encroachment of the subacromial components can be visualized by US, including bouncing of the supraspinatus tendon or bulging of the subdeltoid bursa during shoulder elevation^14^. Our study showed that grade 2 impingement was also associated with BPE and the strength of the association was similar to that of grade 1. Additionally, although many shoulder abnormalities such as calcification and tendinopathy can lead to subacrominal impingement, subdeltoid bursitis was the only variable associated with BPE in static sonography apart from supraspinatus tendon full thickness tear. This result is understandable because the function of the subdeltoid bursa is to decrease friction between the rotator cuff tendons and the acromion. The LHBT, acting as a humeral head depressor, plays a similar role in smoothing shoulder movements^23^. Therefore, thickening of the subdeltoid bursa and BPE might both represent an impaired friction-reducing mechanism. Furthermore, when the subdeltoid bursa is thickened, it would be easier to concomitantly visualize pooling of bursal synovial fluid lateral to the acromion during arm elevation, a grade 2 subacromial impingement.

Our results revealed that grade 3 impingement was associated with BPE but the strength of association was less than that for grade 1 and 2. Compared with Model 1 and 2 (Table 2), the OR of grade 3 impingement with BPE was slightly less in Model 3, the full model including binary variables of shoulder physical findings. However, we found that the only physical finding associated with BPE was shoulder stiffness. This result might indicate that the clinical impingement assessments, like Neer and Hawkins-Kennedy tests, are not as sensitive as dynamic US examinations to grade subacromial impingement. In addition, adhesive capsulitis is known to result in BPE because of an increased effort of the biceps tendon to prevent the humeral head from upward migration during arm elevation^10^. The majority of patients with adhesive capsulitis would be classified as “stiff shoulder” based on the criteria of 50% limitation in shoulder range of motion. Passage of the humeral greater tuberosity underneath the acromion requires abduction up to nearly 90 degrees and the test commonly fails in patients with severely limited shoulder range of motion. Therefore, concurrent presence of adhesive capsulitis and grade 3 impingement would be prevalent among Model 3 and multicollinearity might have mitigated the strength of association between grade 3 impingement and BPE.

Since the LHBT is the first structure checked in a routine shoulder US examination, our study has indisputably important clinical implications. The dynamic subacromial impingement test should be performed when BPE is present because of their strong association. Grading the dynamic excursion of subacromial tissues helps the physician explore functional dyscoordination other than static sonographic markers.

The present study has several limitations. First, we used a cross-sectional design so that the causal relationship between BPE and the dynamic subacromial impingement test could be considered less solid. Second, the prevalence of BPE was higher in our population than that previously reported in the literature. One antecedent study revealed that patients older than 60 years were more likely to have mild or moderate grades of BPE^10^. The main reason could have been that our participants were older and that our data, along with previous observations, all showed a positive association between BPE and increased age^8^,^10^. Third, a change in BPE after treatment of subacromial impingement could not be demonstrated by our study and definitely needs further longitudinal research. Fourth, we were not able to determine the association between BPE and normal rotator cuff tendons in our study. In the face of normal cuff tendons, isolated BPE can still be related to intra-articular pathologies like labral tear or early joint osteoarthritis. These are entities that ultrasound cannot rule out.

## Conclusions

The present study demonstrated that BPE thicker than 1 mm was associated with all grades of ultrasonographic subacromial impingement. The positive association was independent of concurrent static sonographic shoulder aberrations and various physical findings. The dynamic sonographic subacromial impingement test is suggested during routine US examination whenever BPE is present. In order to confirm the causal relationship, future prospective studies are needed to follow up the change in BPE after the treatment of subacromial impingement.

## Additional Information

**How to cite this article**: Chang, K.-V. *et al*. Association of Bicipital Peritendinous Effusion with Subacromial Impingement: A Dynamic Ultrasonographic Study of 337 Shoulders. *Sci. Rep.* **6**, 38943; doi: 10.1038/srep38943 (2016).

**Publisher's note:** Springer Nature remains neutral with regard to jurisdictional claims in published maps and institutional affiliations.

## Figures and Tables

**Table 1:** Baseline characteristics, physical findings and sonographic presentations of shoulders in patients with and without biceps tendon effusion.

	Effusion (−) (N = 191)	Effusion (+) (N = 146)	P value	
Baseline characteristics				
Age (years)	54.9 ± 12.6	60.3 ± 10.8	<0.01*	
Sex				
Female (number, %)	111 (58%)	85 (58%)		
Male (number, %)	80 (42%)	61 (42%)	0.98	
Affected side				
Left	77 (40%)	67 (46%		
Right	114 (60%)	79 (54%)	0.30	
Sonographic presentations (dynamic impingement test)				
Grade 0, no impingement (number, %)	128(67.0%)	39(26.7%)		
Grade 1, pain during shoulder elevation without soft tissue impingement (number, %)	22(11.5%)	45(30.8%)		
Grade 2, pain during shoulder elevation with soft tissue impingement (number, %)	13(6.8%)	31(21.2%)		
Grade 3, pain during shoulder elevation with humeral head upward migration (number, %)	28(14.6%)	31(21.2%)	<0.01*	
Sonographic presentations (static images)				
Subscapularis tendon tear (number, %)	8 (4.2%)	10 (6.8%)	0.28	
Subscapularis tendinopathy (number, %)	23 (12.0%)	23 (15.7%)	0.35	
Subscapularis tendon calcification (number, %)	52(27.2%)	40(27.4%)	0.97	
Subdeltoid bursitis (number, %)	47 (24.6%)	75 (51.3%)	<0.01*	
Supraspinatus tendon full thickness tear (number, %)	10 (5.2%)	29 (19.8%)	<0.01*	
Supraspinatus tendon bursal-sided partial thickness tear (number, %)	1(0.5%)	6 (4.1%)	0.02*	
Supraspinatus tendon articular-sided partial thickness tear (number, %)	13(6.8%)	18(12.3%)	0.08	
Supraspinatus tendinopathy (number, %)	90(47.1%)	72(49.3%)	0.68	
Supraspinatus tendon calcification (number, %)	44(23.0%)	30(20.5%)	0.58	
Infraspinatus tendon tear (number, %)	2(1.1%)	8(2.3%)	0.02*	
Infraspinatus tendinopathy (number, %)	6(3.1%)	12(8.2%)	0.04*	
Infraspinatus tendon calcification (number, %)	8(4.2%)	8(5.5%)	0.58	
Physical findings				
Pain duration (month)	6.8 ± 15.3	7.9 ± 19.8	0.56	
Resting pain (VAS, 10 mm)	2.6 ± 2.2	2.6 ± 2.1	0.92	
Night pain (VAS, 10 mm)	4.0 ± 2.4	4.4 ± 2.2	0.08	
Pain during overhead activities (VAS, 10 mm)	4.9 ± 2.0	5.2 ± 2.0	0.30	
Bicipital groove tenderness (number, %)	97 (50.8%)	84 (57.5%)	0.22	
Speed test (number, %)	63 (32.9%)	63 (43.1%)	0.06	
Yergason’s test (number, %)	30 (15.7%)	25 (17.1%)	0.72	
Empty can test (number, %)	98 (51.3%)	92 (63.0%)	0.03	
Neer test (number, %)	95 (49.7%)	74 (50.6%)	0.86	
Hawkins-Kennedy test (number, %)	110 (57.6%)	94 (64.4%)	0.20	
Painful arc test (number, %)	98 (51.3%)	92 (63.0%)	0.03*	
Range of motion limitation (number, %)	50 (26.2%)	55 (37.7%)	0.02*	

*indicates p < 0.05. VAS: visual analogue scale.

**Table 2:** The association of bicipital peritendinous effusion (BPE) with baseline characteristics static, dynamic sonographic images and physical findings.

	Model 1	Model 2	Model 3
Baseline characteristics			
Age (years)	1.03 (1.01–1.06)*	1.02 (1.00–1.05)	1.02 (0.99–1.04)
Sex			
Female	Reference	Reference	Reference
Male	1.12 (0.68–1.84)	1.04 (0.61–1.78)	1.01 (0.57–1.77)
Affected side			
Left	Reference	Reference	Reference
Right	0.82 (0.53–1.44)	0.67 (0.39–1.16)	0.72 (0.41–1.27)
Sonographic presentations (dynamic impingement test)			
Grade 0 (no impingement)	Reference	Reference	Reference
Grade 1 (pain during shoulder elevation without soft tissue impingement)	6.67 (3.50–12.73)*	6.47 (3.27–12.88)*	6.54 (3.21–13.32)*
Grade 2 (pain during shoulder elevation with soft tissue impingement)	7.45 (3.49–15.90)*	7.28 (3.26–16.26)*	6.93 (3.05–15.76)*
Grade 3 (pain during shoulder elevation with humeral head upward migration)	3.74 (1.97–7.10)*	3.80 (1.87–7.70)*	3.18 (1.48–6.80)*
Sonographic presentations (static images)			
Subscapularis tendon tear	—	0.71 (0.17–2.84)	0.64 (0.15–2.65)
Subscapularis tendon calcification	—	1.08 (0.48–2.45)	1.08 (0.46–2.52)
Subscapularis tendinopathy	—	1.19 (0.64–2.22)	1.14 (0.60–2.18)
Subdeltoid bursitis	—	3.30 (1.84–5.93) *	3.42 (1.88–6.24) *
Supraspinatus tendon full thickness tear	—	4.28 (1.56–11.74)*	4.63 (1.62–13.21)*
Supraspinatus tendon bursal-sided partial thickness tear	—	1.06 (0.35–3.21)	1.11 (0.36–3.41)
Supraspinatus tendon articular-sided partial thickness tear	—	3.95(0.28–54.64)	3.72 (0.27–51.03)
Supraspinatus tendinopathy	—	1.17(0.67–2.05)	1.09 (0.61–1.92)
Supraspinatus tendon calcification	—	1.31 (0.64–2.67)	1.34 (0.64–2.81)
Infraspinatus tendon tear	—	2.23 (0.32–15.38)	2.61 (0.35–19.23)
Infraspinatus tendinopathy	—	1.03 (0.23–4.52)	0.92 (0.20–4.20)
Infraspinatus tendon calcification	—	0.77 (0.19–3.02)	0.87 (0.21–3.57)
Physical findings			
Bicipital groove tenderness	—	—	0.92 (0.49–1.71)
Speed test	—	—	1.18 (0.62–2.28)
Yergason’s test	—	—	0.68 (0.32–1.48)
Empty can test	—	—	1.21 (0.66–2.21)
Neer test	—	—	0.74 (0.38–1.43)
Hawkins-Kennedy test	—	—	0.89 (0.46–1.71)
Painful arc test	—	—	1.34 (0.70–2.56)
Shoulder stiffness	—	—	1.89 (1.02–3.51)*

The value was expressed by an odds ratio with 95% confidence interval. *Indicates p < 0.05.
